# *Lactobacillus paragasseri* HM018 derived from breast milk ameliorates hyperlipidemia in high-cholesterol rats by modulating bile acid metabolism

**DOI:** 10.3389/fmicb.2025.1599931

**Published:** 2025-06-04

**Authors:** Chunyu Yao, Xianping Li, Mi Tang, Lu Liu, Xiaoqian Cai, Xueping Yuan, Jufeng Hu, Junying Zhao, Weicang Qiao, Yue Zhang, Lijun Chen

**Affiliations:** ^1^School of Biological Engineering, Dalian Polytechnic University, Dalian, China; ^2^National Engineering Research Center of Dairy Health for Maternal and Child, Beijing Sanyuan Foods Co. Ltd., Beijing, China; ^3^Beijing Engineering Research Center of Dairy, Beijing Technical Innovation Center of Human Milk Research, Beijing Sanyuan Foods Co. Ltd., Beijing, China; ^4^Key Laboratory of Dairy Science, Ministry of Education, Food Science College, Northeast Agricultural University, Harbin, China; ^5^College of Life Sciences, Inner Mongolia University, Hohhot, China

**Keywords:** hyperlipidemia, breast milk-derived probiotics, *Lactobacillus paragasseri*, gut microbiota, bile acid metabolism, cholesterol efflux

## Abstract

**Introduction:**

Hyperlipidemia, a prevalent metabolic disorder with rising global incidence, has become a major public health concern. Probiotics allow for a mild intervention strategy for hyperlipidemia management that has garnered increasing attention.

**Methods:**

In this study, we investigated the therapeutic effects and underlying mechanisms of *Lactobacillus paragasseri* HM018 in hypercholesterolemic rats.

**Results and discussion:**

We established three dosage groups (2.5 × 10^8^, 5 × 10^8^, and 1.5 × 10^9^ CFU/rat), demonstrating that HM018 significantly reduced high-fat diet-induced serum total cholesterol, triglycerides, and low-density lipoprotein cholesterol levels, while ameliorating gut microbiota dysbiosis and decreasing the *Firmicutes*/*Bacteroidetes* ratio. Our transcriptomic analysis revealed that HM018 markedly upregulated *Apoa1* expression both in the ileum and liver, while enhancing *Abcg5*/*Abcg8* gene expression to promote *β*-sitosterol efflux. Concurrently, hepatic *Cocs2/3* and *Cish* gene expression was downregulated, attenuating their inhibitory effects on hormonal and glucagon signaling, thereby improving glucose and lipid metabolism. Metabolomic profiling further indicated that HM018 significantly altered bile acid composition by modulating gut microbiota-mediated bile acid metabolism. In conclusion, *Lactobacillus paracasei* HM018 could ameliorate hyperlipidemia through multiple pathways, including gut microbiota modulation, hepatic lipid/glucose/bile acid metabolism improvement, and intestinal cholesterol efflux gene expression enhancement.

## 1 Introduction

Hyperlipidemia is a highly prevalent metabolic disorder worldwide (Su et al., 2022), characterized by elevated levels of total cholesterol (TC), triglycerides (TG), and low-density lipoprotein cholesterol (LDL-C), as well as reduced high-density lipoprotein cholesterol (HDL-C). The primary causes include high carbohydrate or fat intake, lack of exercise, and delayed intervention and treatment, leading to increased blood lipid levels (Khanali et al., 2023). Consequently, it triggers related diseases such as cardiovascular disease (CVD) and nonalcoholic fatty liver disease (NAFLD) (James et al., 2018; Roth et al., 2020). The prevalence and mortality rates of hyperlipidemia and its complications are increasing in various countries, making it a significant public health issue (James et al., 2018). In particular, CVDs induced by elevated blood lipid levels have become a major burden on healthcare services (Roth et al., 2020). Statins are commonly used drugs for treating hyperlipidemia (Lamb, 2020) and are particularly effective in reducing LDL-C levels. However, there is considerable interindividual variability in their lipid-lowering efficacy, which may be related to the composition of the gut microbiota (Jia et al., 2021; Hu et al., 2024).

The “gut microbiota” is a complex and dynamic community of microorganisms in the human intestine (Ross et al., 2024). The impact of a high-fat diet (HFD) on the structure of the gut microbiota has been well-documented. Dysbiosis of the gut microbiota leads to increased intestinal permeability, elevated levels of pro-inflammatory cytokines, insulin resistance, glucose intolerance, and a series of other issues (Howard et al., 2022). Gut microbes can influence blood lipid levels through metabolites such as short-chain fatty acids, lipopolysaccharide, and bile acids (Jia et al., 2021). Gut microbiota is an effective target for the prevention and treatment of metabolic syndromes. For example, *Blautia producta*, screened from human gut microbiota, inhibits lipid accumulation in cells and reduces lipid levels in the blood and liver of HFD-fed mice (Xu W. et al., 2023). *Enterococcus faecium* B6, isolated from obese children, promotes hepatic lipid accumulation, inflammation, and fibrosis through the bioactive metabolite tyramine, thereby contributing to NAFLD (Wei et al., 2024). Intervention with *Lactococcus lactis* subsp. Cremoris in Western diet-fed mice reduces hepatic steatosis and inflammation, as well as serum cholesterol and body mass index (Naudin et al., 2020).

Probiotics, particularly strains of the genus *Lactobacillus*, are reportedly significantly efficient in ameliorating dyslipidemia (Gao et al., 2021), although substantial functional heterogeneity exists among different strains (Patnode et al., 2021). In this study, we isolated a *Lactobacillus* strain, *Lactobacillus paragasseri*, from human milk. Based on the microbial taxonomic criteria (ANI < 95% and isDDH <70% for novel species designation), this strain represents a novel species reclassified from *L. gasseri* [exhibiting ANI values of 93.4–93.7% and isDDH values of 53.1–54.1% versus the reference strain ATCC 33323; (Tanizawa et al., 2018)], retaining an incompletely characterized mechanistic potential in improving hyperlipidemia. Through oral gavage administration, we systematically investigated the lipid-lowering mechanisms of human milk-derived *L. paragasseri* HM018 in high-fat diet-induced hyperlipidemic rats. Experimental results demonstrated that HM018 intervention significantly attenuated diet-induced serum triglyceride, total cholesterol, and low-density lipoprotein level increase. Integrated gut microbiota composition, fecal metabolomics, and ileal/hepatic transcriptomics analyses revealed the multifaceted regulatory effects of this probiotic on lipid metabolism.

## 2 Materials and methods

### 2.1 *L. Paragasseri* HM018 and culture medium

*L. paragasseri* HM018 is derived from healthy breast milk and is presently conserved at the China General Microbiological Culture Collection Center (CGMCC No. 19749). *L. paragasseri* HM018, isolated from breast milk, was preserved at the National Engineering Research Center for Dairy Health (Beijing, China). The strain was inoculated in De Man–Rogosa–Sharpe broth (Luqiao Co., Ltd., Beijing, China) at 37°C for 18 h and subcultured twice at a 1% inoculation rate to ensure bacterial activity. Cellular biomass was harvested via refrigerated centrifugation (4°C, 6,000 rpm, 15 min). The supernatant was removed, and the pellet was washed twice with sterile 0.01 M phosphate-buffered saline (PBS; 0.01 M concentration, sterile filtered, Solarbio). Finally, the bacterial suspension was adjusted to low-dose (2.5 × 10^8^ colony-forming units [CFU] mL-1), medium-dose (5 × 10^8^ CFU mL-1), and high-dose (1.5 × 10^9^ CFU mL-1) concentrations for oral gavage.

### 2.2 Rat modeling and feeding

All animal procedures adhered to the Guidelines for the Care and Use of Laboratory Animals established by Beijing Union University andreceived approval from the Animal Ethics Committee of Beijing Union University (JCZX11-2306-1, Beijing, China).

Fifty adult male Sprague–Dawley rats (body weight 200 ± 20 g; Vital River Laboratories, Beijing, China), raised under specific pathogen-free (SPF) conditions, were acclimatized for one week under standard conditions with a 12-h light/dark cycle. Throughout the acclimatization period, food and water were freely available. Subsequently, the rats were randomly assigned to five experimental groups as follows: (1) a normal diet (ND) control group receiving standard chow and 2 mL of sterile PBS; (2) an HFD model group receiving an HFD and 2 mL of sterile PBS; and three treatment groups (3–5) receiving the HFD supplemented with varying concentrations of *L. paragasseri* HM018: (3) the low-dose group (LPL) received 2 mL of a 2.5 × 10^8^ CFU/mL suspension; (4) the medium-dose group received 2 mL of a 5 × 10^8^ CFU/mL suspension; and (5) the high-dose group (LPH) received 2 mL of a 1.5 × 10^9^ CFU/mL suspension, all administered via gavage. All experimental diets were provided by Ke’ao Xieli Feed Co., Ltd. (Beijing, China).

After six weeks of intervention, following a 12-h fast after the final gavage, fecal samples were collected and incubated at 25 ± 1°C for 60 min. Subsequently, the rats were euthanized, and blood samples were collected, and Serum was obtained through centrifugation of blood samples at 3,000 × g for 15 min at 4°C. Tissue specimens (liver, epididymal adipose, ileal segments) were rapidly cryopreserved in liquid nitrogen vapor phase and maintained at −80°C.

### 2.3 Biochemical analysis

Serum biomarkers, including TG, TC, LDL-C, HDL-C, glucose, and glycated hemoglobin (GHb), were quantified using commercial assay kits (Nanjing Jiancheng Bioengineering Institute, China) following manufacturer protocols.

### 2.4 Hematoxylin and eosin (H&E) staining

Liver tissue samples from rats were fixed in 4% paraformaldehyde at room temperature for 24 h. Subsequently, the samples were dehydrated, embedded in paraffin, and sectioned into 5 μm slices. After dewaxing, the tissue sections were stained with hematoxylin and eosin (H&E) for histological observation.

### 2.5 Gene sequencing analysis of 16S rRNA

Using the OMEGA Mag-bind DNA Kit, genomic DNA was isolated from samples. The V3-V4 region of the 16S rRNA gene was then amplified with specific barcoded primers 341F (CCTAYGGGRBGCASCAG) and 806R (GGACTACNNGGGTATCTAAT). Polymerase chain reaction (PCR) products were analyzed using 2% agarose gel electrophoresis and subsequently purified using a Quant-iT PicoGreen dsDNA Assay Kit. Based on the preliminary quantification results from electrophoresis, the recovered PCR products were quantified using a fluorescence quantification system on a microplate reader (BioTek, FLx800). Library construction was performed using an Illumina TruSeq Nano DNA LT Library Prep Kit. The quality of the constructed libraries was assessed using an Agilent Bioanalyzer 2,100 and Promega QuantiFluor, followed by sequencing. Raw sequencing data were processed to remove primer sequences using Cutadapt, and quality control and annotation were performed using QIIME 2 with the SILVA database (version 138). Alpha and beta diversity indices were calculated using QIIME 2.

### 2.6 HPLC analysis

Fecal samples were collected from 10 rats per group (ND, HFD, and LPH groups; n = 30 total). Samples underwent liquid nitrogen flash-freezing and were maintained at −80°C Untargeted metabolomic analysis was performed at Shanghai Applied Protein Technology Co., Ltd. (Shanghai, China) using an ultra-high-performance liquid chromatography (UHPLC) system (1,290 Infinity II, Agilent Technologies) coupled with a quadrupole time-of-flight mass spectrometer (AB Sciex TripleTOF 6,600).

Fecal samples (50 mg) were homogenized with 400 μL methanol/acetonitrile (1:1, v/v) via vortex mixing, followed by sequential processing: 30-min sonication at 5°C (40 kHz), 30-min incubation at-20°C, and centrifugation (1,400 × g, 20 min, 4°C). The resulting supernatant was vacuum-dried and reconstituted in 120 μL acetonitrile/water (1:1, v/v). For quality assurance, 10 μL QC sample was introduced prior to final 5-min sonication/centrifugation, with processed supernatant ultimately transferred to LC–MS vials for analysis.

Chromatographic separation was performed on a Waters Acquity UPLC BEH Amide column (100 mm × 2.1 mm, 1.7 μm) employing hydrophilic interaction liquid chromatography (HILIC). The mobile phase consisted of (A) 25 mM ammonium acetate/ammonium hydroxide aqueous solution and (B) acetonitrile, with the following gradient profile: initial 95% B (0–0.5 min), linear gradient to 65% B (0.5–7.5 min), stepwise reduction to 40% B (7.5–7.6 min), isocratic hold (7.6–8.6 min), immediate return to 95% B (8.6–8.7 min), and 3-min column re-equilibration (8.7–11.7 min). The chromatographic system was maintained at 0.3 mL/min flow rate and 40°C.

Instrument parameters were configured as follows: ion source gases (60 psi), curtain gas (30 psi), 600°C source temperature, ±5,500 V ion spray voltage. The method employed 35 V (±15 eV) collision energy, ±60 V declustering potential, 4 Da isotope exclusion, and 10-cycle candidate ion-monitoring acquisition. Raw data were converted into mzXML format using ProteoWizard MSConvert. Peak detection, alignment, and annotation were performed using the XCMS workflow, with metabolites identified by matching accurate mass (m/z error <10 ppm) and MS/MS spectra against an in-house reference database.

### 2.7 Transcriptome analysis

Total RNA was extracted from the liver tissues of rats in the three groups (ND, HFD, and LPH) using the TRIzol reagent. RNA purity was evaluated by measuring the A260/A280 ratio using a Nanodrop ND-2000 spectrophotometer (Thermo Scientific, USA), and RNA integrity was assessed using an Agilent Bioanalyzer 4,150 system (Agilent Technologies, CA, USA). Subsequently, paired-end libraries were prepared using the ABclonal mRNA-seq Lib Prep Kit (ABclonal, China) following manufacturer protocols. Sequencing was conducted across dual platforms: Illumina Novaseq 6,000 and MGISEQ-T7 systems.

Sequencing reads were preprocessed using Trimmomatic (adapter removal/quality trimming). Genome alignment was performed using HISAT2. DESeq2 (v1.40.2) implemented in R was used to determine differentially expressed genes (criteria: |log2FC| > 1, FDR < 0.05). Enrichment analysis was performed using GO and KEGG pathways (clusterProfiler v4.10.0). Statistical significance was set at *p* < 0.05.

### 2.8 Gene expression profiling by real-time quantitative PCR

Rat liver and ileum tissue samples were collected. Total RNA was extracted using RNA-easy Isolation Reagent (R701-01, Vazyme, China), and RNA was reverse-transcribed into cDNA using the HiScript II Q RT SuperMix for qPCR (+gDNA wiper) (R223-01, Vazyme, China) kit. Subsequently, using ChamQ SYBR Master Mix (Q311-02, Vazyme, China) as the reaction system, real-time fluorescence quantitative PCR (RT-PCR) was carried out on an Applied Biosystems 7,500 Real-Time PCR System to detect the expression levels of the target genes (*Abcg5, Abcg8, Socs2*, and *Cish*). The primer sequences are as follows: for *Abcg5*, the forward primer is 5′-GTCCTTCAGCGTCAGCAACC-3′ and the reverse primer is 5′ -ATGGTCTGGCCACTCTCGAT-3′; for *Abcg8*, the forward primer is 5′-CACCCTAGACTCTAACTCCA-3′ and the reverse primer is 5′-GGAGCACTGGATAGTATTGG-3′ (Jang et al., 2022); for *Socs2*, the forward primer is 5′-GAACCACGCTGTCAAACT-3′ and the reverse primer is 5′-CTCCCACTCAGACTACCTATT-3′ (Dang et al., 2020); for *Cish*, the forward primer is 5′- TACCTCCGGGGATCTGGTTG −3′ and the reverse primer is 5′- CACGGGTGGTTTTGACTGAC -3′. Gadph (forward primer: 5′-GAAACCTGCCAAGTATGA-3′, reverse primer: 5′-GCTGTAGCCGTATTCATT-3′) was used as the internal reference gene for normalization analysis.

### 2.9 Statistical analysis

Graphical representations, including histograms, box plots, and heatmaps, were generated using Origin software (Version 2024, OriginLab, MA, USA). Statistical analyses were conducted using SPSS (Version 27.0.1, IBM, USA), employing one-way analysis of variance followed by Tukey’s post-hoc test for multiple comparisons. Data are presented as mean ± standard deviation (SD), with statistical significance set at *p* < 0.05. Additionally, bivariate correlations were evaluated using Spearman’s rank-order correlation method.

## 3 Results

### 3.1 HM018 ameliorates dyslipidemia and hepatic abnormalities in HFD-fed rats

**Figure 1:**
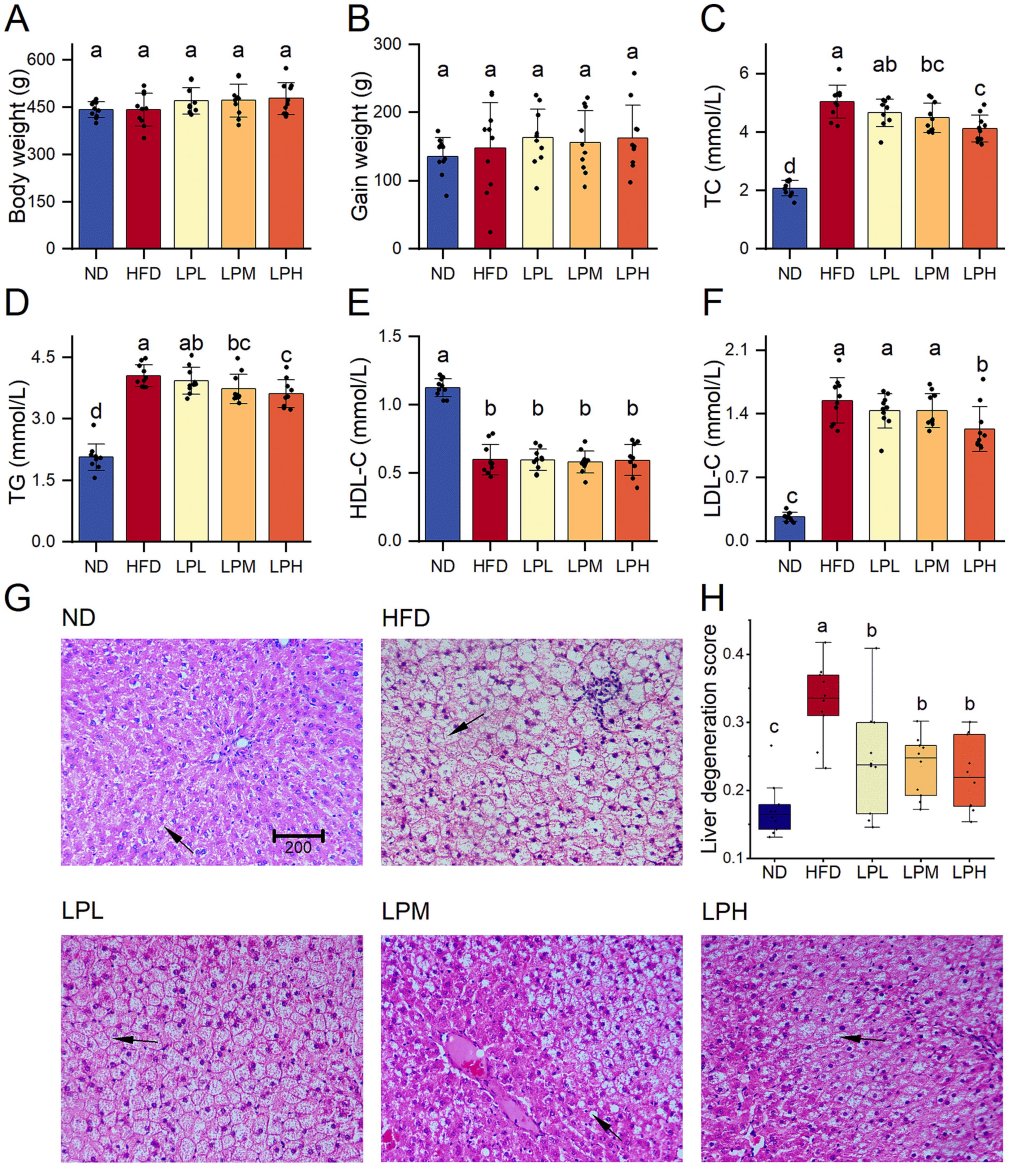
Effects of *Lactobacillus paragasseri* HM018 on dyslipidemia in rats. **(A)** Body weight. **(B)** Gain weight. **(C)** Serum total cholesterol (TC). **(D)** Serum triglycerides (TG). **(E)** High-density lipoprotein cholesterol (HDL-C). **(F)** Low-density lipoprotein cholesterol (LDL-C). **(G)** Hematoxylin and eosin staining of pathological sections of adipose tissue. Scale bar: 200 μm. **(H)** Quantitative analysis of the hepatic adipocyte area. ND: Normal diet; HFD: High-fat diet; LPL: Low-dose intervention group (2.5 × 10^8^ CFU/rat); LPM: Medium-dose intervention group (5 × 10^8^ CFU/rat); LPH: High-dose intervention group (1.5 × 10^9^ CFU/rat); CFU: Colony-forming units. Significance groupings (*p* < 0.05) identified by Tukey’s test are represented through differential lowercase alphabetic annotations above corresponding boxplots within individual panels.

After 6 weeks on a high-fat diet, serum TG and TC levels were notably increased in HFD group. In the probiotic intervention groups with varying doses, these parameters showed dose-dependent improvements. Additionally, the HFD group demonstrated significantly elevated LDL-cholesterol levels compared to the ND group. But was significantly reduced after probiotic intervention. However, HDL-C level, which is associated with lipid utilization, significantly decreased after HFD, and there was no change in its concentration after the intervention with HM018. Compared to the ND group, body weight, fasting glucose, and GHb levels showed no statistically significant variations across the experimental cohorts (Supplementary Figure S1). Histological analysis showed that HFD led to fat accumulation, and the ratio of the area of liver adipocytes in the HFD group was significantly higher than that in other groups. In summary, the abnormal elevation of key lipid markers (TG, TC, and LDL-C) confirmed the successful establishment of the HFD-induced rat model, while probiotic intervention significantly ameliorated dyslipidemia (Figure 1).

### 3.2 HM018 ameliorates the dysbiosis induced by an HFD

To elucidate the impact of HM018 supplementation on intestinal microbiome composition, 16S rRNA gene sequencing was conducted across thirty fecal specimens. A total of 3,914 amplicon sequence variants (ASVs) were obtained, with 1,189, 801, and 2,168 ASVs identified in the HFD model, experimental LPH, and control ND groups, respectively. The numbers of unique ASVs in the ND, HFD, and LPH groups were 1,423, 359, and 218, respectively (Supplementary Figure S2A).

**Figure 2:**
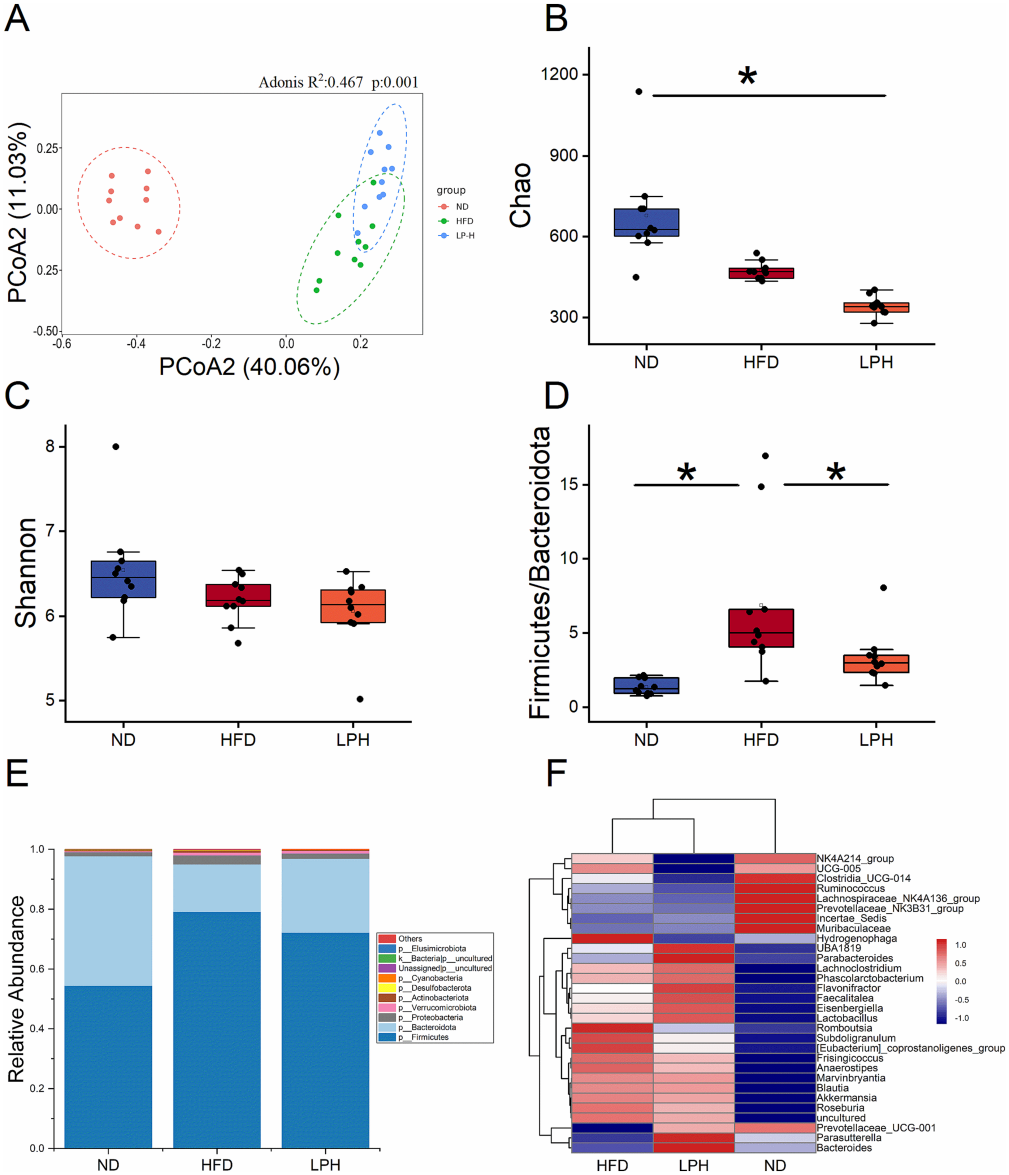
Effects of *Lactobacillus paragasseri* HM018 intervention on gut microbiota in high-fat diet-fed rats. **(A)** Principal coordinate analysis (PCoA) plot based on the Bray–Curtis distance matrix, illustrating structural differences in the gut microbiota among the three groups. **(B)** Chao1 and **(C)** Shannon indexes reflecting alpha diversity changes across groups. **(D)** Firmicutes-to-Bacteroidetes ratio in the gut microbiota. **(E)** Relative distribution of gut microbiota across groups. **(F)** Heatmap showing variations in the genus-level microbial composition. ND: Normal diet; HFD: High-fat diet; LPH: *L. paragasseri* HM018 intervention group. *p < 0.05, **p < 0.01 indicate statistical significance.

Principal coordinates analysis revealed a significant separation in the gut microbiota structure between the HFD (green) and ND (red) groups after HFD feeding. Following HM018 intervention, the LPH group (blue) also exhibited a distinct microbiota composition compared to the HFD group. These results demonstrate significant HFD-induced perturbations in gut microbiota composition and that HM018 intervention modulated these changes (Figure 2A).

Alpha diversity analysis revealed that the HFD significantly reduced microbial richness (Chao1 and ACE indices) in the HFD group, and this reduction was further exacerbated after probiotic intervention (Figure 2B; Supplementary Figure S2B). While Shannon and Simpson diversity indices showed no statistically significant variations in the HFD cohort (*p* > 0.05), both metrics demonstrated consistent decreasing trends. In the dose-dependent analysis, the Chao1 and ACE indices gradually decreased with increasing probiotic dosage, whereas the Shannon and Simpson indices significantly increased (Figure 2C, Supplementary Figure S2C).

Phylum-level analysis identified five predominant bacterial taxa: Firmicutes, Bacteroidetes, Verrucomicrobia, Proteobacteria, and Actinobacteria. Compared with the ND and LPH groups, the relative abundance of Firmicutes was significantly increased in the HFD group, whereas that of Bacteroidetes was significantly decreased. The HFD significantly elevated the Firmicutes-to-Bacteroidetes ratio, which was significantly reduced after HM018 intervention (Figures 2D,E).

At the genus level, the heatmap analysis clustered the microbiota into three groups. *Muribaculaceae*, *Clostridia*_UCG-014, and *Lachnospiraceae*_NK4A136_group were the dominant genera in the ND group. Whereas, HFD significantly suppressed the abundance of *Muribaculaceae* and significantly increased the abundances of *Blautia*, *Hydrogenophaga*, *Subdoligranulum*, *Romboutsia*, *Akkermansia*, and [*Eubacterium*]*_coprostanoligenes*_group. In the LPH group, the abundances of *Parasutterella*, *Bacteroides*, *Parabacteroides*, and *Prevotellaceae*_UCG − 001 significantly increased after HM018 intervention (*p* < 0.05). In addition, UBA1819, *Eisenbergiella*, *Lachnoclostridium*, *Roseburia*, and *Phascolarctobacterium* abundances also increased (*p* > 0.05) (Figure 2F).

### 3.3 HM018 alters fecal bile acid composition

**Figure 3:**
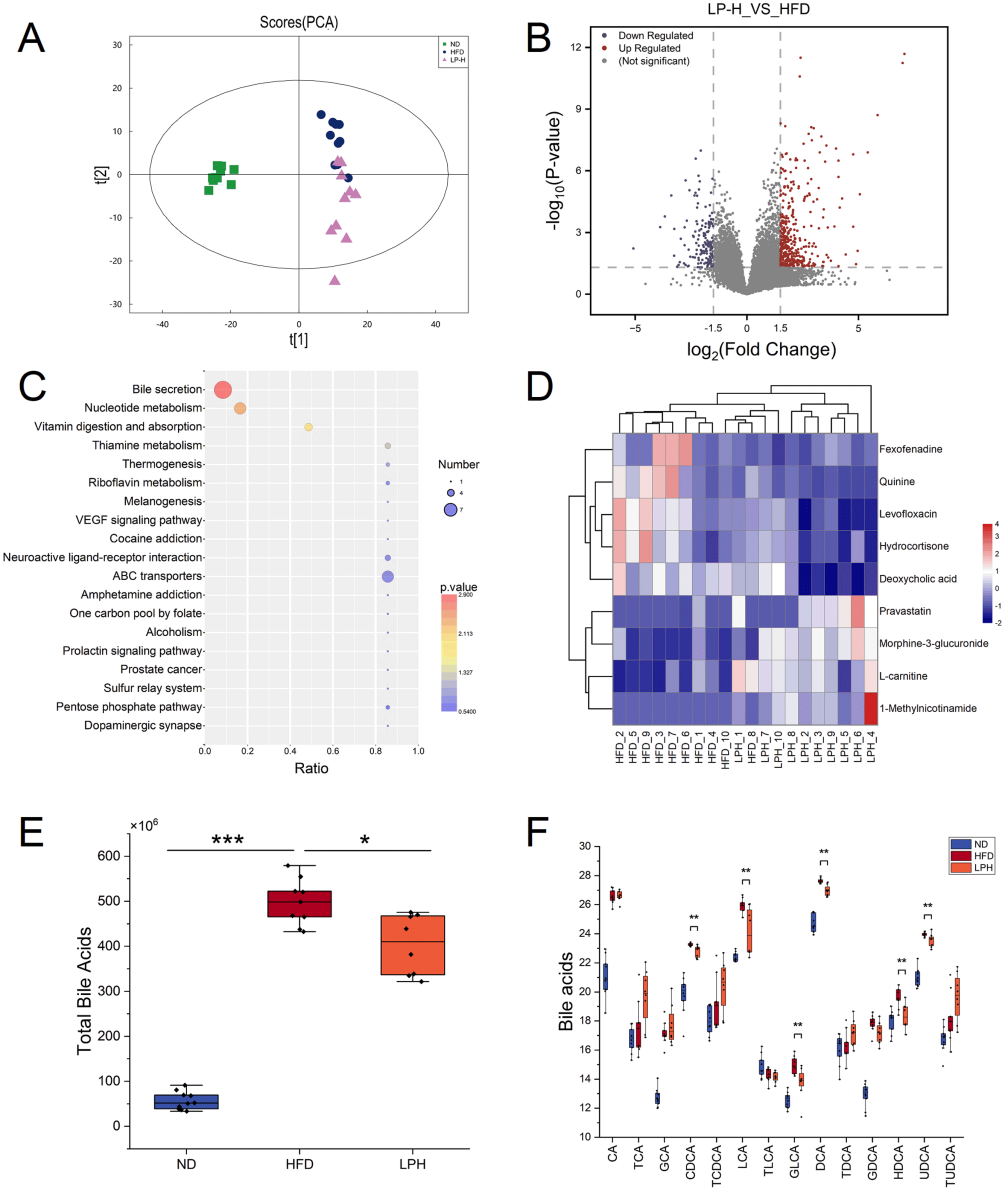
Effects of *Lactobacillus paragasseri* HM018 intervention on fecal metabolites in high-fat diet (HFD)-fed rats. **(A)** Principal component analysis (PCA) showing the overall differences in fecal metabolites between the intervention (LPH) and model (HFD) groups. **(B)** Volcano plot of differential metabolites in the LPH group compared to the HFD group, with red and blue dots representing significantly upregulated and downregulated metabolites, respectively. **(C)** Kyoto Encyclopedia of Genes and Genomes pathway enrichment analysis of differential metabolites, with significantly enriched pathways displayed as a dot plot. **(D)** Heatmap of differential metabolites involved in bile acid metabolism, showing relative abundance changes of key bile acids in the LPH and HFD groups. **(E)** Differences in total bile acid content in feces before and after intervention. **(F)** Changes in key bile acids before and after *L. paragasseri* HM018 intervention, including cholic acid (CA), taurocholic acid (TCA), glycine-conjugated cholic acid (GCA), chenodeoxycholate (CDCA), taurochenodeoxycholic acid (TCDCA), lithocholic acid (LCA), taurolithocholic acid (TLCA), glycolithocholic acid (GLCA), deoxycholic acid (DCA), taurodeoxycholic acid (TDCA), glycine-conjugated deoxycholic acid (GDCA), hyodeoxycholic acid (HDCA), ursodeoxycholic acid (UDCA), and tauroursodeoxycholic acid (TUDCA). *p < 0.05, **p < 0.01 indicate statistical significance.

To delineate gut microbiota-metabolite interactions in hyperlipidemic models, we implemented LC–MS-based untargeted metabolomics profiling across three experimental cohorts (ND, HFD and LPH) Partial least squares-discriminant analysis (PLS-DA) revealed distinct clustering among the three groups, with significant separation between the LPH and HFD groups after HM018 intervention (Figure 3A). Comparative metabolomic analysis identified 1,134 differentially expressed metabolites (DEMs) in the HFD group relative to ND controls, including 288 downregulated and 847 upregulated metabolites. In contrast, the LPH group showed 166 upregulated and 90 downregulated metabolites compared to the HFD group (Figure 3B).

KEGG pathway enrichment analysis indicated that the differential metabolites were significantly enriched in pathways such as bile secretion, nucleotide metabolism, vitamin digestion and absorption, and thiamine metabolism (Figure 3C). Key metabolites involved in bile secretion included morphine-3-glucuronide, pravastatin, 1-methylnicotinamide, L-carnitine, fexofenadine, deoxycholic acid, quinine, hydrocortisone, and levofloxacin (Figure 3D). Additionally, bile acid homeostasis was significantly altered across the three groups: total bile acid levels were markedly elevated after HFD feeding but significantly reduced following HM018 intervention (Figure 3E). Compared to the HFD group, the LPH group exhibited significant downregulation of free bile acids, such as lithocholic acid (LCA), hyodeoxycholic acid (HDCA), chenodeoxycholate (CDCA), ursodeoxycholic acid (UDCA), and deoxycholic acid (DCA), and upregulation of conjugated bile acids such as taurocholic acid (TCA), glycine-conjugated cholic acid (GCA), taurochenodeoxycholic acid (TCDCA), taurodeoxycholic acid (TDCA), and tauroursodeoxycholic acid (TUDCA) (Figure 3F).

### 3.4 HM018 intervention improves lipid efflux and intestinal permeability

**Figure 4:**
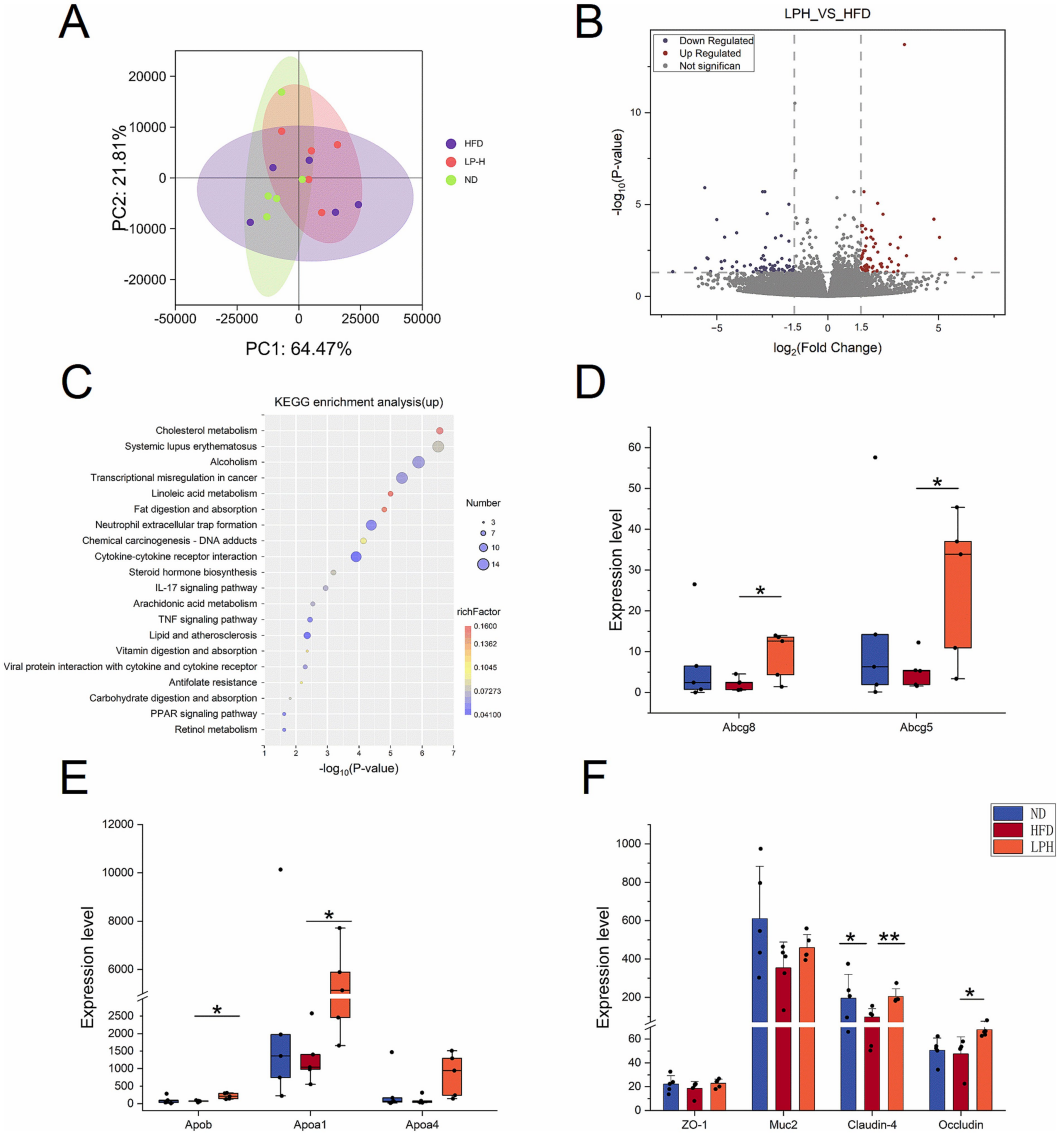
Effects of *Lactobacillus paragasseri* HM018 intervention on the ileal transcriptome in hyperlipidemic rats. **(A)** Principal component analysis (PCA) showing transcriptomic differences in ileal samples among the control (ND), model (HFD), and intervention (LPH) groups. **(B)** Volcano plot of differentially expressed genes in the ileum, with red and blue dots representing significantly upregulated and downregulated genes, respectively. **(C)** Kyoto Encyclopedia of Genes and Genomes (KEGG) pathway enrichment analysis of upregulated genes, with enriched pathways displayed as a dot plot. **(D)** Expression levels of ATP-binding cassette transporters 5/8 (*Abcg5/8*) in ileal samples from the three groups. **(E)** Expression differences of apolipoprotein A-1 (*Apoa1*), apolipoprotein A-4 (*Apoa4*), and apolipoprotein B (*Apob*) among the three groups. **(F)** Transcriptional levels of intestinal tight junction components (*ZO-1, Claudin-4, Occludin*) and the goblet cell mucin 2(*MUC2*) in three treatment cohorts. *p < 0.05, **p < 0.01 indicate statistical significance.

To elucidate host gene regulation by HM018-derived metabolites, we implemented transcriptomic sequencing of ileal specimens from three rat groups (15 samples total). Transcriptomic PCA clustering showed LPH group profiles were more similar to ND than HFD (Figure 4A). The LPH-HFD comparison yielded 213 significant DEGs, including 117 upregulated and 96 downregulated genes (Figure 4B).

KEGG pathway enrichment analysis demonstrated that the upregulated genes were significantly enriched in cholesterol metabolism, linoleic acid metabolism, fat digestion and absorption, vitamin digestion and absorption, arachidonic acid metabolism, steroid hormone biosynthesis, carbohydrate digestion and absorption, retinol metabolism, PPAR signaling pathway, and glutathione metabolism (Figure 4C). Notably, the cholesterol efflux-related genes *Abcg5* and *Abcg8* were markedly upregulated post-intervention, and this result was consistently verified by qPCR (Figure 4D; Supplementary Figure S3). Additionally, key regulators of lipid metabolism, including HDL-associated *Apoa1* and intestinal lipid transporter *Apoa4*, demonstrated significant transcriptional upregulation (Figure 4E).

Downregulated genes were primarily enriched in pathways such as systemic lupus erythematosus, transcriptional dysregulation in cancer, alcoholism, and neutrophil extracellular trap formation (Supplementary Figure S3). To evaluate intestinal barrier integrity, we analyzed the expression of tight junction components (*ZO-1, Claudin-4, Occludin*) and the goblet cell marker *Muc2*. Both *Occludin* and *Muc2* were significantly upregulated after HM018 intervention (Figure 4F).

### 3.5 HM018 intervention ameliorates hepatic glucose and lipid metabolism

**Figure 5:**
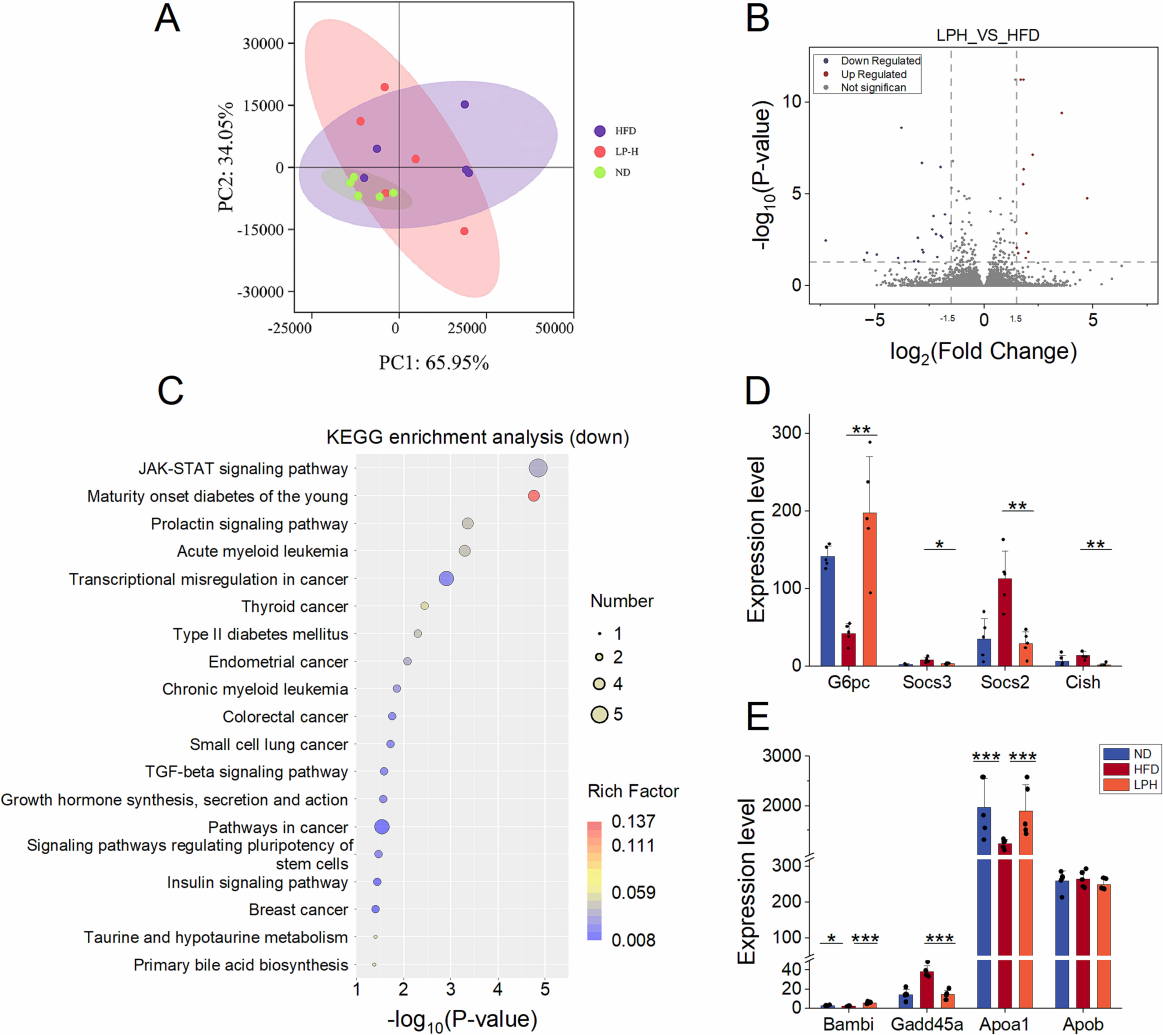
Effects of *Lactobacillus paragasseri* HM018 intervention on hepatic transcriptomics in hyperlipidemic rats. **(A)** Principal component analysis (PCA) revealed the differences in the transcriptomes of the ileum samples. **(B)** Volcano plot of differentially expressed genes in the liver. **(C)** Kyoto Encyclopedia of Genes and Genomes (KEGG) pathway enrichment analysis of downregulated genes, with significantly enriched pathways displayed as a dot plot. **(D)** Expression levels of genes related to lipogenesis and metabolism in the liver, including glucose-6-phosphatase (*G6PC*), suppressor of cytokine signaling 2/3 (*Socs2* and *Socs3*), and cytokine-inducible SH2-containing protein (*Cish*). **(E)** Expression differences of BMP and activin membrane-bound inhibitor (*Bambi*), growth arrest and DNA damage-inducible alpha (*Gadd45a*), apolipoprotein A-1 (*Apoa1*), and apolipoprotein B (*Apob*) among the three groups (*p < 0.05, **p < 0.01).

To explore the effect of HM018 on rat livers, we performed RNA-seq analysis on liver samples from three groups: ND, LPH, and HFD. PCA revealed that the LPH and HFD groups exhibited more dispersed distributions than the ND group, indicating that both HFD and HM018 intervention significantly altered the hepatic transcriptomic profiles (Figure 5A). Further analysis identified 24 upregulated and 35 downregulated genes in the LPH group compared to those in the HFD group (Figure 5B). Additionally, Pathway enrichment profiling identified key lipid metabolism-related signaling cascades: JAK–STAT, TGF-*β*, FoxO transcriptional regulation, insulin signaling, adipocytokine communication, and diabetes pathways (Figure 5C; Supplementary Figure S4).

Among the genes related to lipogenesis, although not statistically significant, their transcriptional levels generally showed a downward trend after HM018 intervention (Supplementary Figure S4). Meanwhile, analysis of glucose metabolism-related genes revealed that HM018 intervention significantly upregulated glucose-6-phosphatase (*G6pc*) expression, while downregulating suppressor of cytokine signaling 2/3 (*Socs2* and *Socs3*) and cytokine-inducible SH2-containing protein (*Cish*). Quantitative PCR (QPCR) results further confirmed that the expression of *Socs2* and *Cish* in the LPH group was significantly reduced, validating this regulatory trend (Figure 5D; Supplementary Figure S4). Notably, HM018 intervention restored the expression of BMP and activin membrane-bound inhibitor (*Bambi*), growth arrest and DNA damage-inducible alpha (*Gadd45a*), and *Apoa1* to levels comparable to those in the ND group. In contrast, *Apob* (low-density lipoprotein metabolism regulator) exhibited no significant expression differences across experimental groups (Figure 5E). These results suggest that HM018 may ameliorate hepatic metabolic disorders by multi-target regulation of key genes involved in glucose and lipid metabolism.

### 3.6 Integrated analysis of microbiome, metabolome, and transcriptome correlations

**Figure 6:**
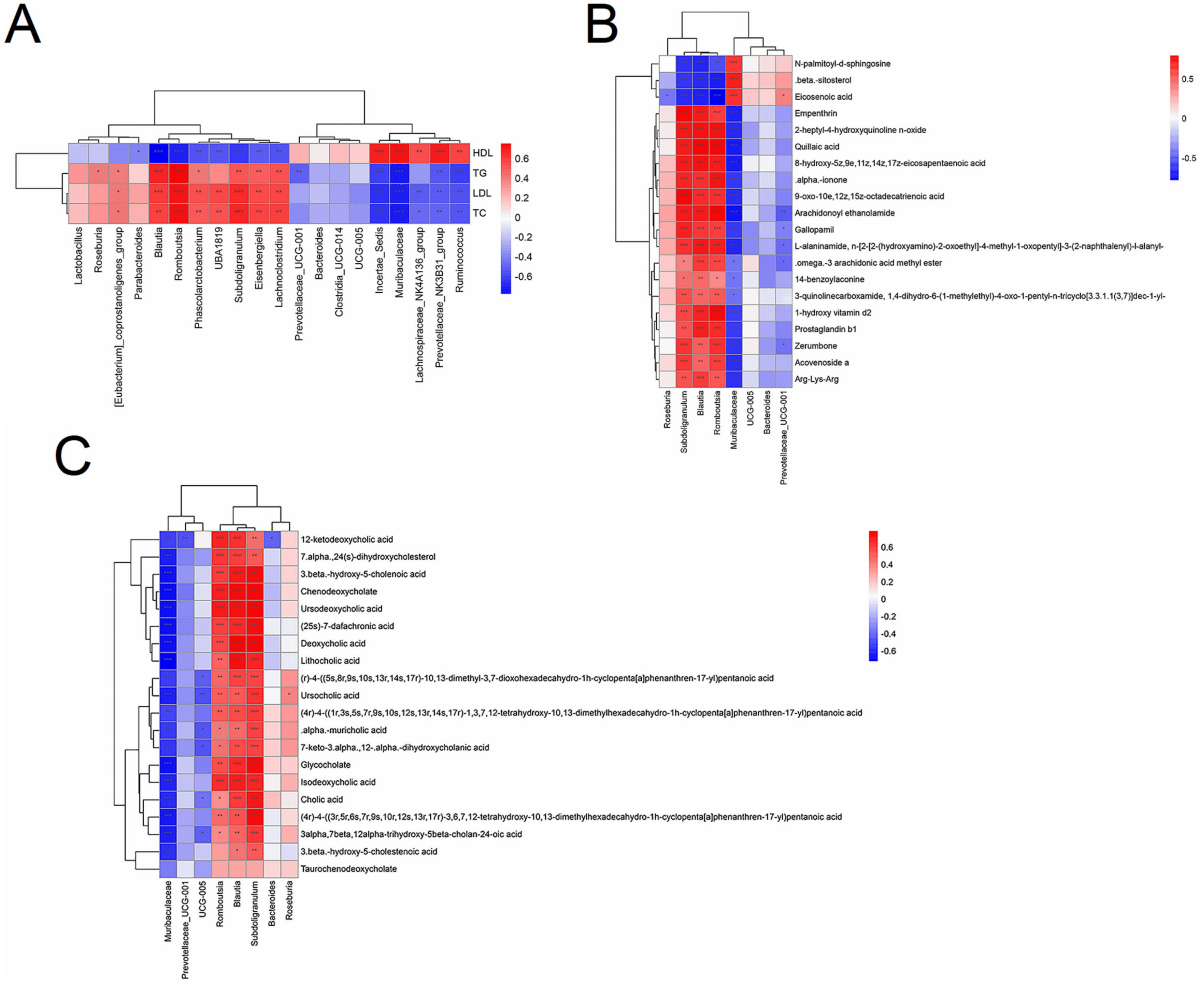
Correlation analysis between lipid profiles and the microbiome/metabolome. **(A)** Spearman’s correlation analysis between lipid profiles and gut microbiota at the genus level. **(B)** Spearman’s correlation analysis between gut microbiota and differential metabolites. **(C)** Spearman’s correlation analysis between gut microbiota and differential bile acids and their derivatives. The color gradient ranges from blue (negative correlation) to red (positive correlation). *p < 0.05, **p < 0.01, and ***p < 0.001 indicate significant correlations.

We first conducted a joint analysis of the four significantly altered bacterial phyla (Firmicutes, Bacteroidetes, Verrucomicrobia, and Proteobacteria) post-HM018 intervention using lipid profiles across the three experimental groups. The results showed that, except for Bacteroidetes, the relative abundances of Firmicutes, Verrucomicrobia, and Proteobacteria were inversely related to HDL levels, while demonstrating positive correlations with triglyceride, total cholesterol, and LDL levels (Supplementary Figure S5A). Genus-level analysis identified positive correlations between Blautia, Romboutsia, Subdoligranulum abundances and dyslipidemia severity scores, whereas *Muribaculaceae*, *Incertae-Sedis*, *Lachnospiraceae_NK4A136_group*, *Ruminococcus*, and *Prevotellaceae_UCG-001* were significantly correlated with improved hyperlipidemia (Figure 6A).

In the joint analysis of differential metabolites and lipid parameters, *β*-sitosterol, eicosenoic acid, and N-palmitoyl-D-sphingosine were strongly negatively correlated with TG, TC, and LDL (r < −0.7; *p* < 0.05), but strongly positively correlated with HDL (*r* > 0.7; *p* < 0.05). Increased concentrations of these metabolites were associated with better prognostic outcomes, whereas other metabolites were significantly enriched in hypercholesterolemic rats (|*r*| > 0.7) (Supplementary Figure S5B).

A combined analysis of differential metabolites and bacterial genera demonstrated that *Muribaculaceae*, a dominant genus in the ND group, showed strong positive correlations with eicosenoic acid, β-sitosterol, and N-palmitoyl-D-sphingosine, but negative correlations with other compounds (|r| > 0.7). In the intervention group, the increased abundance of *Prevotellaceae_UCG-001* weakly correlated with eicosenoic acid and *ω*-3 arachidonic acid methyl ester (0.5 > |r| > 0.3). Notably, the dominant genera in the model group, *Blautia* and *Subdoligranulum*, displayed moderate negative correlations with β-sitosterol (−0.6 > r > −0.7) and positive correlations with compounds such as empenthrin (r > 0.3) (Figure 6B).

Furthermore, HM018 intervention significantly altered the composition of the bile acid pool in rats. Joint analysis of differential bile acids and their derivatives with bacterial genera revealed that *Muribaculaceae* was negatively correlated with most bile acids, whereas *Blautia*, *Subdoligranulum*, and *Romboutsia* showed significant positive correlations with bile acids (Figure 6C). These findings suggest that HM018 may ameliorate dyslipidemia by modulating the interaction between the gut microbiota and bile acid metabolism.

## 4 Discussion

This study demonstrates that *L. paragasseri* HM018 could significantly improve HFD-induced hyperlipidemia in rats, with marked serum TG, TC, and LDL level as well as adipocyte size reductions post-intervention (*p* < 0.05). With an increasing HM018 intervention dose, serum TC and TG levels displayed a gradient downward trend, and when the dose reached 10^9^ CFU/rat, the TC, TG and LDL levels were significantly reduced (*p* < 0.05).

HFD alters gut microbiota composition, whereas probiotic intervention remodels the microbial structure and alleviates dysbiosis (Smirnova et al., 2022; Kappel et al., 2023). HFD increases the Firmicutes-to-Bacteroidetes ratio, which is associated with metabolic disorders such as dyslipidemia (Yasuzawa et al., 2022; Xu et al., 2023a), and elevates the abundance of Clostridiales within Firmicutes (Hewady et al., 2024), consistent with our findings. Obesity is closely linked to inflammation, as fat accumulation and localized inflammation reduce the expression of tight junction proteins (e.g., *claudin-1, occludin*, and *ZO-1*) (Chelakkot et al., 2018; Acciarino et al., 2024), thereby increasing intestinal permeability and triggering inflammation (Kim et al., 2024). HM018 intervention notably improved intestinal permeability.

Cholesterol, a key precursor of bile acid synthesis, is converted into primary bile acids in hepatocytes via the classical (mediated by CYP7A1) and alternative (mediated by CYP27A1) pathways. Subsequently, bile acid-CoA synthase and bile acid-CoA:amino acid N-acyltransferase catalyze the conjugation of primary bile acids with taurine or glycine to form bile salts stored in the gallbladder (Collins et al., 2023; Xu et al., 2023b). During the fed state, BAs are released into the gastrointestinal tract, where gut microbiota hydrolyze conjugated bile acids into free primary BAs via bile salt hydrolase (BSH), followed by 7*α*-dehydroxylase-mediated 7α-dehydroxylation to generate secondary BAs (DCA and LCA) (Long et al., 2017). High-cholesterol, low-fiber diets upregulate hepatic bile acid synthesis and elevate systemic bile acid levels (Chaudhari et al., 2021), further promoting lipid absorption and obesity. HM018 intervention significantly reduced total bile acid levels. HFD enriches gut microbiota with BSH and 7α-dehydroxylase activities, particularly *Blautia, Eubacterium*, and *Clostridium* species, leading to intestinal deoxycholic acid (DCA) accumulation and significant conjugated bile acid (T-α-MCA, T-*β*-MCA, TCA, TUDCA, and GCA) suppression, thereby establishing a pro-obesity bile acid metabolic disorder (Lin et al., 2019; Liu et al., 2021). External interventions could effectively modulate this dysbiosis by suppressing BSH-producing *Clostridium* while promoting *Bacteroidetes* and *Akkermansia* proliferation. This microbial remodeling reduces primary and increases conjugated bile acids, ultimately improving dyslipidemia and hepatic steatosis (Zhang et al., 2024). Recent studies have shown that a ketogenic diet inhibits gut microbial bile salt hydrolase, elevating circulating TDCA and TUDCA levels to reduce energy absorption, promote weight loss, and lower fasting glucose (Li et al., 2024). Elevated circulating TDCA, glycodeoxycholic acid, and glycoursodeoxycholic acid levels, along with reduced fecal DCA and UDCA levels, correlate with improved weight and glycemic control in patients with obesity (Chaudhari et al., 2021). Moreover, in the *Lactobacillus plantarum* HT121-associated hyperlipidemia improvement, serum TG, TC, and LDL levels significantly positively correlated with the relative abundance of the *Blautia* and *Clostridium* genera (Li et al., 2020). Our results demonstrate that HM018 intervention suppresses *Blautia* and *Clostridium* strains expressing BSH and 7α-dehydroxylase activities while promoting *Bacteroidetes* proliferation. This modulation significantly reduces free bile acid levels (including LCA, HDCA, CDCA, UDCA, and DCA; *p* < 0.05) and upregulates conjugated bile acids (TCA, GCA, TCDCA, TDCA, and TUDCA; 0.05 < *p* < 0.1), thereby effectively improving hyperlipidemia.

Reverse cholesterol transport (RCT), a critical process for eliminating excess cholesterol from the peripheral tissues to the liver, is driven by apolipoprotein A-I (*ApoaI*). Synthesized in the liver and intestine, *ApoaI* is a major component of HDL (Duong et al., 2008; Luo et al., 2020) and facilitates cholesterol efflux for biliary excretion (Liu et al., 2025). Mutations, enzymatic modifications, or metabolic alterations in *ApoaI* reduce HDL quality and contribute to dyslipidemia and CVDs (Bhale and Venkataraman, 2022). Transcriptomic analysis revealed that HM018 significantly upregulated *ApoaI* expression in the liver and ileum.

Under HFD conditions, the transintestinal cholesterol excretion (TICE) pathway, which is independent of biliary secretion and facilitated by *Abcg5/8*, becomes crucial in the elimination of cholesterol via feces (Luo et al., 2020; Xu et al., 2023b). Genetic variants of *Abcg5/8* elevate plasma *β*-sitosterol and LDL cholesterol (Tada et al., 2022; Alenbawi et al., 2024). Our correlation analysis showed increased fecal β-sitosterol and cholesterol sulfate levels in the intervention group, suggesting that HM018 upregulates ileal *Abcg5/8* transcription to enhance sterol excretion via TICE, thereby alleviating hyperlipidemia.

Several hepatic genes are associated with lipogenesis and metabolism. Among these, the upregulation of *GADD45A* promotes subcutaneous fat deposition and obesity (You et al., 2023b). *Gadd45a* knockout mice exhibited enhanced lipolysis, improved energy utilization, and increased resistance to HFD-induced obesity (You et al., 2023a). *Bambi* knockout exacerbates HFD-induced metabolic disorders, including hepatic steatosis, glucose intolerance, and insulin resistance (Chen X. et al., 2021), whereas *Bambi* overexpression suppresses obesity (Weber et al., 2023). Following HM018 intervention, the transcriptional level of *Gadd45a* was significantly downregulated, whereas that of *Bambi* was upregulated, indicating that HM018 improved insulin sensitivity and restored glucose metabolism. Additionally, HFD upregulated the transcriptional expression of *Cish*, *Socs2*, and *Socs3*. Previous studies have demonstrated that *Cish*-deficient mice fed an HFD exhibit reduced body weight and fat mass, along with improved insulin sensitivity (Chen G. et al., 2021; Lynch et al., 2024). However, glucose tolerance remains unchanged, accompanied by increased glucagon levels and sensitivity (Naser et al., 2022; Xiao et al., 2022). *Socs3* is upregulated in obesity and inhibits leptin and insulin signaling (Palanivel et al., 2012), while its downregulation reduces hepatic lipid levels in obese Sprague–Dawley rats (Jiang et al., 2024). *Socs2* upregulation promotes lipogenesis by negatively regulating growth hormone signaling and suppressing lipolysis (Yang et al., 2013). After HM018 intervention, hepatic expression of *Cish*, *Socs2*, and *Socs3* was significantly downregulated, thereby attenuating their inhibitory effects on hormone and glucagon signaling (Gaudy et al., 2010; Palanivel et al., 2012; Zadjali et al., 2012). *G6pc*, a rate-limiting enzyme in glycogenolysis and gluconeogenesis, exhibited suppressed transcription in the HFD group. However, HM018 intervention restored *G6pc* expression to control levels. These findings suggest that HM018 may enhance glucagon sensitivity to promote glucose production, thereby modulating the utilization of glucose and lipids This study demonstrates that HM018 can significantly improve the lipid profile in high-cholesterol rats. However, it is important to note that there are species-specific differences in bile acid composition and binding between humans and rodents. Therefore, the lipid-lowering effects of HM018 in humans still need to be explored through further clinical studies (Wahlström et al., 2016).

In summary, *L. paragasseri* HM018 alleviated HFD-induced dyslipidemia by restoring hepatic and intestinal metabolic homeostasis. It modulated gut microbiota and bile acid metabolism to promote cholesterol excretion via the RCT and TICE pathways. Additionally, HM018 enhanced insulin sensitivity and glucose metabolism by modulating the expression of lipid-related genes.

## 5 Conclusion

This study demonstrates that *L. paragasseri* HM018 significantly ameliorates HFD-induced hyperlipidemia in rats. HM018 not only effectively regulates lipid profiles and remodels gut microbiota composition but also improves intestinal permeability. Furthermore, it modulates bile acid homeostasis, promotes RCT, and enhances cholesterol excretion. At the transcriptional level, HM018 significantly improves insulin sensitivity and alleviates glucose metabolism disorders. These findings provide a solid theoretical foundation for the potential application of HM018 as a therapeutic intervention in hyperlipidemia and related metabolic diseases.

## Data Availability

The data presented in the study are deposited in the Mendeley Data repository (https://data.mendeley.com), under the following DOI: 10.17632/r75rntjrnn.1.
