# Supplementary material for: Lactobacillus paragasseri HM018 derived from breast milk ameliorates hyperlipidemia in high-cholesterol rats by modulating bile acid metabolism
Source: Front Microbiol. 2025 Jun 4;16:1599931. doi: 10.3389/fmicb.2025.1599931 (PMC12174092; doi:10.3389/fmicb.2025.1599931)
Supplement: Supplementary file 1 [file Data_Sheet_1.docx]

Supplementary Material





**Supplementary Figure 1.** Effects of *Lactobacillus* *paracasei* HM018 on dyslipidemia in rats. **(A)** Glycated hemoglobin. **(B)** Fasting blood glucose. ND: Normal diet; HFD: High-fat diet; LPL: Low-dose intervention group (2.5×10⁸ CFU/rat); LPM: Medium-dose intervention group (5×10⁸ CFU/rat); LPH: High-dose intervention group (1.5×10⁹ CFU/rat); CFU: Colony-forming units.





**Supplementary Figure 2.** Effects of *Lactobacillus paracasei* HM018 intervention on gut microbiota in high-fat diet-fed rats. **(A)** Venn diagram of ASVs distribution across three experimental groups. **(B)** Simpson index and **(C)** Chao index reflecting alpha diversity changes across groups. **(D)** Firmicutes-to-Bacteroidetes ratio in the gut microbiota. ND: Normal diet; HFD: High-fat diet; LPL: Low-dose intervention group (2.5×10⁸ CFU/rat); LPM: Medium-dose intervention group (5×10⁸ CFU/rat); LPH: High-dose intervention group (1.5×10⁹ CFU/rat). Significance groupings (*p* < 0.05) identified by Tukey’s test are represented through differential lowercase alphabetic annotations above corresponding boxplots within individual panels.





**Supplementary Figure 3.** Effects of HM018 intervention on the ileal transcriptome in hyperlipidemic rats. **(A)** Kyoto Encyclopedia of Genes and Genomes (KEGG) pathway enrichment analysis of downregulated genes, with significantly enriched pathways visualized as a dot plot. **(B)** Relative expression levels of*Abcg5* (ATP-binding cassette subfamily G member 5) in ileal samples from the three groups. **(C)** Relative expression levels of *Abcg8* (ATP-binding cassette subfamily G member 8) in ileal samples from the three groups. **p* < 0.05, ***p*< 0.01 indicate statistical significance.





**Supplementary Figure 4.** Effects of HM018 intervention on hepatic transcriptomics in hyperlipidemic rats. **(A)** Kyoto Encyclopedia of Genes and Genomes (KEGG) pathway enrichment analysis of upregulated genes, with significantly enriched pathways visualized as a dot plot. **(B)**Expression differences of *Fasn* (Fatty Acid Synthase), *Acly* (ATP Citrate Lyase), *Acaca* (Acetyl-CoA Carboxylase Alpha), *Elovl5* (Elongation of Very Long Chain Fatty Acids Protein 5), and *Faah* (Fatty Acid Amide Hydrolase) in the liver. **(C)** Relative expression levels of *Cish* (cytokine-inducible SH2-containing protein) in ileal samples from the three groups. **(D)** Relative expression levels of suppressor of *Socs2* ( cytokine signaling 2) in liver samples from the three groups. **p* < 0.05, ***p* < 0.01 indicate statistical significance.





**Supplementary Figure 5.** Correlation analysis between lipid profiles and the microbiome/metabolome. **(A)** Spearman’s correlation analysis between lipid profiles and gut microbiota at the phylum level. **(B)** Spearman’s correlation analysis between lipid profiles and differential metabolites. The color gradient ranges from blue (negative correlation) to red (positive correlation). **p* < 0.05, ***p* < 0.01, and ****p* < 0.001 indicate significant correlations.
